# Artificial Intelligence-Integrated Biosensors for Antimicrobial Resistance Detection and Surveillance: A Review and Future Perspectives for Global Biosecurity

**DOI:** 10.7759/cureus.98098

**Published:** 2025-11-29

**Authors:** Olabisi P Lawal, Innocent J Opara, Ayodele Ayo-ige, Ndidi A Eboh, Uchechukwu Cos-Ibe, Kwesi Akonu Adom Mensah Forson, Elijah Kordieh Mensah, Ololade F Olaitan, Enoch Nii-Okai, Alfred Yeboah, Nazeem Gabriels, Aliyu O Olaniyi

**Affiliations:** 1 Medical Laboratory Science, University of Benin, Benin City, NGA; 2 Computer Information Systems, Prairie View A&M University, Prairie View, USA; 3 Infectious Diseases, Yale University, New Haven, USA; 4 Nursing, Stark State College, North Canton, USA; 5 Computer Science, Georgia State University, Atlanta, USA; 6 Biology, University of Virginia, Charlottesville, USA; 7 Environmental Studies, University of Montana, Missoula, USA; 8 Information Systems, University of Utah, Salt Lake City, USA; 9 Mining and Minerals Engineering, Michigan Technological University, Houghton, USA; 10 Emergency Medicine, Stockport National Health Service (NHS) Foundation Trust, Stockport, GBR; 11 Geriatrics, Stepping Hill Hospital, Stockport, GBR

**Keywords:** antimicrobial resistance (amr), artificial intelligence (ai), biosensors, machine learning (ml), one health

## Abstract

Antimicrobial resistance (AMR) poses a critical threat to global health, undermining the efficacy of modern medicine. The escalating global epidemic of AMR jeopardizes the efficacy of contemporary medicine and undermines health systems globally. The swift, precise, and scalable identification of resistance determinants is essential for containment and stewardship initiatives; yet, existing surveillance techniques are constrained by time, expense, and accessibility. Recent advancements in biosensor technology and artificial intelligence (AI) provide a revolutionary approach to decentralized, intelligent AMR monitoring. This review consolidates recent advancements in biosensor platforms-encompassing electrochemical, optical, piezoelectric, paper-based, and nanomaterial-based modalities-and their incorporation with AI and machine learning techniques for improved detection, signal interpretation, and predictive analytics. This study investigates the utilization of hybrid systems in clinical, veterinary, and environmental settings under the One Health surveillance framework. The research also examines the integration of AI-enabled biosensors within digital and Internet of Things (IoT) frameworks, emphasizing its capacity to produce real-time, data-intensive insights for public health decision-making. Critical analysis is conducted on key problems, including sensor repeatability, data scarcity, algorithmic transparency, and regulatory adaptation, in conjunction with socioeconomic and ethical considerations. The report delineates prospective avenues for research, policy, and implementation, highlighting open data standards, equitable access, and interdisciplinary collaboration. These breakthroughs collectively indicate the emergence of AI-driven biosensing networks, which provide predictive, adaptive, and globally coordinated AMR surveillance.

## Introduction and background

Antimicrobial resistance (AMR) poses one of the most pressing global health challenges of the 21st century, threatening the effectiveness of essential medicines and undermining human, animal, and environmental health systems. The World Health Organization (WHO) estimates that bacterial resistance directly causes approximately 1.27 million deaths annually and contributes to nearly five million more. Without urgent and coordinated intervention, AMR could result in 10 million deaths each year by 2050 and impose an estimated economic burden exceeding USD 100 trillion. As this crisis transcends clinical boundaries, a unified One Health approach encompassing human, animal, and environmental sectors is crucial for effective detection, surveillance, and mitigation [1].

Although traditional culture-based methods for pathogen identification and susceptibility testing remain accurate, they typically require 24-72 hours to yield results. While polymerase chain reaction (PCR) and next-generation sequencing (NGS) have improved turnaround times, they remain resource-intensive, laboratory-bound, and unsuitable for decentralized or field-based applications. The rapid evolution of resistance mechanisms and the global spread of pathogens demand rapid, portable, and data-driven diagnostic tools capable of providing actionable insights in real time [2].

Biosensors represent a transformative class of analytical devices designed to convert biological interactions into measurable signals through the use of biorecognition elements and physicochemical transducers. Over the past two decades, biosensor technology has advanced from basic electrochemical systems to sophisticated nanomaterial-based and microfluidic platforms. Their compact design, affordability, and high sensitivity make them promising tools for AMR detection in clinical, veterinary, and environmental settings. However, when applied to complex biological matrices, biosensors often encounter challenges such as signal variability, noise interference, limited multiplexing, and complex data interpretation [3].

The integration of artificial intelligence (AI) and machine learning (ML) into biosensing represents a paradigm shift toward intelligent, adaptive, and predictive diagnostic systems. AI enables multidimensional data processing, pattern recognition, and enhanced signal discrimination beyond conventional human analysis. ML models facilitate continuous learning and performance optimization as new resistance data become available, giving rise to smart biosensors capable of autonomous decision-making and remote data transmission [4]. Real-time, decentralized AMR monitoring through AI-driven biosensor analytics can support dynamic feedback loops linking detection, surveillance, and response systems. Such innovations hold the potential to identify resistance hotspots, guide antibiotic stewardship, and inform global health policy [5].

Despite this progress, significant barriers persist. Conventional AMR diagnostics are constrained by speed, scalability, and accessibility, while emerging AI-enabled biosensing systems face challenges related to data scarcity, interpretability, and regulatory validation. Addressing these limitations requires an integrated understanding of biosensor design, computational modeling, and policy frameworks that support safe, transparent, and equitable deployment.

This review critically examines the convergence of biosensor technologies, AI, and AMR surveillance within the context of global health and biosecurity goals. It highlights technological progress, identifies existing challenges, and explores strategies for scalable, equitable, and policy-aligned implementation consistent with the WHO Global Action Plan on AMR and the One Health framework. While the primary focus is on bacterial AMR detection, the discussion expands to encompass One Health surveillance, underscoring the role of AI-enabled biosensing in shaping the future of global biosecurity.

## Review

AMR detection: current landscape

AMR detection is essential to worldwide infectious disease surveillance and clinical decision-making. Resistant pathogen identification is crucial for treatment, epidemic containment, and policymaking. Despite breakthroughs in microbiological and molecular diagnostics, technological, temporal, and infrastructural constraints restrict rapid, scalable AMR surveillance [6].

Conventional methods

The clinical gold standard for AMR detection is culture-based tests. Disk diffusion, broth microdilution, and automated susceptibility testing (VITEK, Phoenix, and MicroScan) produce accurate phenotypic profiles of bacterial resistance. These procedures take 24-72 hours and require live bacterial cultures, which is inconvenient for acute infections or low-resource settings with limited laboratory capacity [7]. To overcome timing constraints, molecular approaches have been developed to directly detect genetic resistance determinants. PCR techniques quickly identify resistance genes, including blaCTX-M, mecA, and vanA, whereas quantitative and multiplex PCR detect numerous targets. NGS and whole genome sequencing (WGS) provide unprecedented resolution for analyzing resistance genes, plasmid transfer, and evolutionary processes. These methods have transformed AMR research and monitoring, but their reliance on advanced hardware, specialized workers, and extensive bioinformatics pipelines restricts their accessibility and scalability, especially in field or point-of-care settings [8].

Molecular and culture-based phenotypic tests, including matrix-assisted laser desorption/ionization time-of-flight mass spectrometry (MALDI-TOF MS), microcalorimetry, and microfluidic growth inhibition systems, are being used for quicker detection. These methods infer resistance using biochemical or biophysical signals and computer analysis. Despite gaining popularity, they still struggle to identify closely related resistance mechanisms or detect low-abundance targets [9].

Challenges and limitations

Conventional platforms for AMR detection face several significant challenges. They are often time-inefficient, with multiday turnaround periods that delay clinical interventions and hinder rapid epidemic response. The high cost and infrastructure requirements further limit their utility, as these assays typically demand regulated laboratory conditions, complex equipment, and skilled personnel. In addition, conventional systems exhibit limited portability, making them unsuitable for decentralized testing in communities, farms, or environmental settings. Data fragmentation is another critical issue, as discrete outputs from surveillance databases slow the integration of epidemiological intelligence. Together, these limitations underscore the urgent need for innovative detection systems that combine precision, speed, and accessibility to address the accelerating threat of AMR [10,11].

Need for innovation

Diagnostics for AMR in the future must go beyond lab boundaries. To satisfy global AMR surveillance targets, the One Health architecture necessitates quick, portable, data-rich detection equipment for ongoing monitoring and real-time reporting. To transform unprocessed inputs into actionable knowledge, these systems need to integrate biological sensing with advanced data analytics. AI and biosensors have the potential to democratize data collection, decentralize AMR detection, and connect diagnostic results to surveillance networks. This change is essential to a paradigm of AMR monitoring that is more global, predictive, and responsive [12].

Biosensors for AMR detection

Biosensors are essential analytical instruments for the evolving diagnosis of AMR. Biosensors use a physicochemical transducer and a biological recognition factor to provide quantifiable signals, combining biotechnology, materials science, and analytical chemistry. They are crucial for clinical, veterinary, and environmental AMR surveillance because of their rapid, sensitive, and on-site identification of microbial illnesses and resistance determinants [13].

Types of biosensors

Electrochemical biosensors are the most investigated AMR detectors. These devices evaluate electrical qualities including current, voltage, and impedance caused by biological interactions between the target analyte and the biorecognition element. Electrochemical platforms are downsized for point-of-care applications and have high sensitivity, low detection limits, and low cost. Examples of sensors include β-lactamase activity detection, mecA gene hybridization, and impedance spectroscopy for antibiotic susceptibility testing [12]. Fluorescence, surface plasmon resonance (SPR), and colorimetric optical biosensors convert biomolecular interactions into optical signals. They detect without labels and monitor in real time. Fluorescence-based technologies can detect resistance-indicating nucleic acid hybridization or enzyme-mediated events, whereas SPR-based sensors quantify antibiotic-protein interactions and resistance dynamics. Despite their precision, optical systems require complex optics and controlled conditions, which may limit field adoption [14]. Piezoelectric and acoustic biosensors use mass or viscoelastic changes to detect targets. Quartz crystal microbalance (QCM) sensors assess frequency shifts related to bacterial adhesion or enzymatic activity to detect resistant infections. These label-free methods use mechanical reaction patterns to distinguish resistant and susceptible strains in real time [15]. Because of their portability, affordability, and suitability for point-of-care and resource-constrained scenarios, paper-based and microfluidic biosensors are widely used. Capillary flow or microchannel devices allow for multiplexed assays on a chip or strip. Microfluidic systems for rapid phenotypic antibiotic susceptibility testing and paper-based colorimetric instruments for β-lactamase detection enable decentralized AMR detection and field surveillance [16].

**Figure 1:**
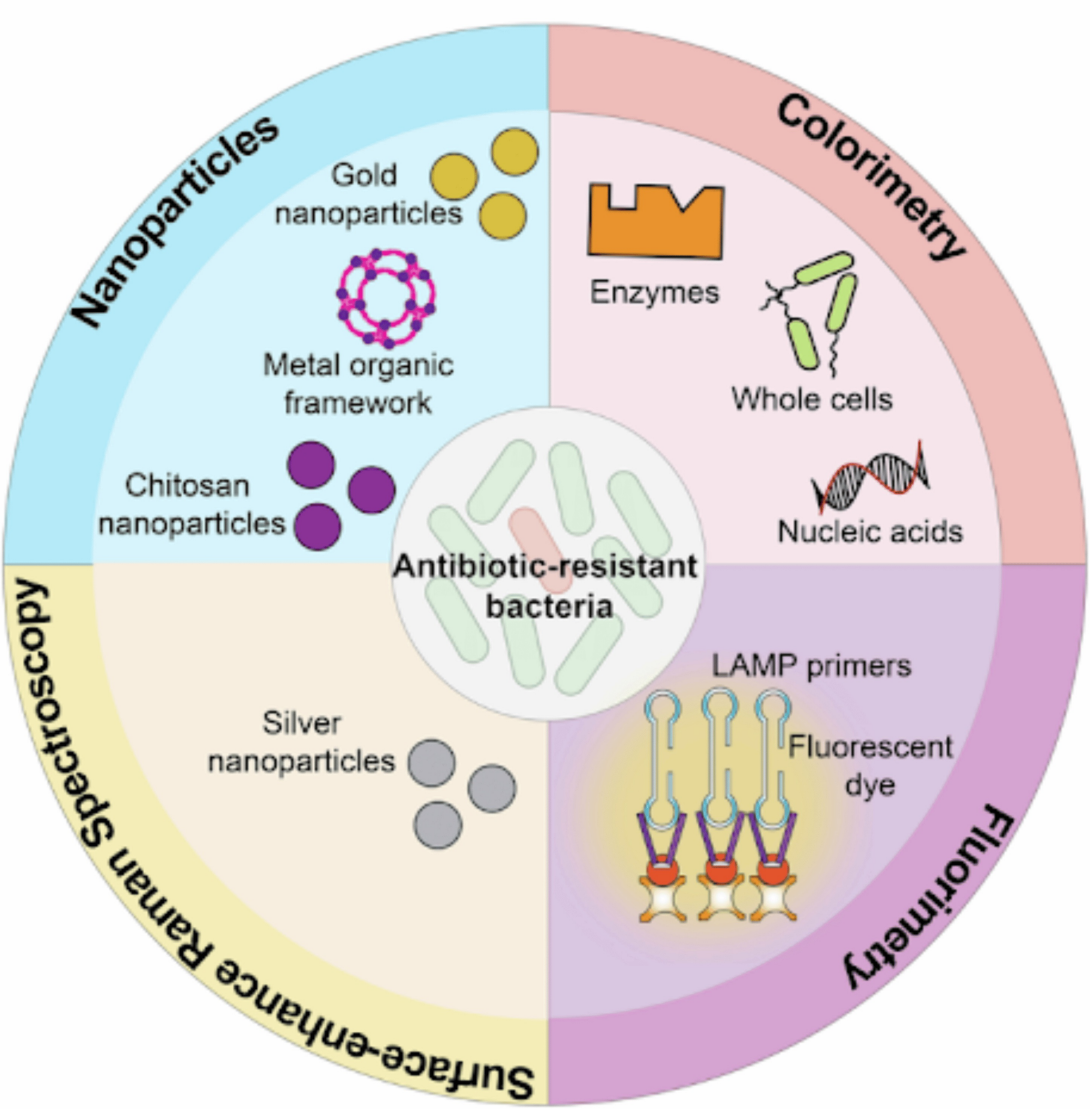
Paper-based sensors using detection methods (colorimetry, fluorometry, surface-enhanced Raman spectroscopy, and nanoparticles) and various targets for identification of AMR bacterial species. AMR: antimicrobial-resistant The image is adapted from [18] (Creative Commons Attribution-NonCommercial-NoDerivatives 4.0 International (CC BY-NC-ND 4.0))

Nanomaterial-based biosensors enhance sensitivity. Nanostructures like gold nanoparticles, quantum dots, graphene, carbon nanotubes, and magnetic nanobeads boost transduction signals and enable single-molecule or ultralow-concentration detection. For label-free nucleic acid recognition, graphene-based electrochemical sensors offer high electron transfer efficiency, whereas quantum dot-based fluorescent sensors can detect resistance genes through fluorescence resonance energy transfer (FRET). These platforms can be integrated into portable and wireless formats and perform well analytically [17]. Figure 1 shows paper-based sensors employing different detection methods including colorimetry, fluorometry, surface-enhanced Raman spectroscopy (SERS), and nanoparticles, to identify various AMR bacterial species.

Biorecognition elements

Biosensor functionality relies on biorecognition to selectively interact with the target analyte. Known recognition components include antibodies, aptamers, enzymes, peptides, and whole-cell biosensors. High-specificity antibody-based systems may have stability and batch variability issues. SELEX (systematic evolution of ligands by exponential enrichment)-selected synthetic oligonucleotides called aptamers have comparable affinity, stability, reusability, and modification ease. Catalytic activity, such as β-lactamase or oxidase, is used in enzyme-based biosensors to measure resistance phenotypes. Functional screening and environmental monitoring of AMR dissemination benefit from the use of whole-cell biosensors, which employ genetically engineered microbial hosts to express reporter genes in response to antibiotic exposure [18].

Performance parameters

Biosensor efficacy depends on sensitivity, specificity, limit of detection (LOD), reaction time, and mobility. Early resistance identification requires high sensitivity and low LOD, especially in low-bacterial-load samples. Specificity distinguishes resistant and susceptible strains or resistance genes. Portability and cost-effectiveness influence feasibility in decentralized or low-resource areas, while response time determines operational utility in clinical and outbreak scenarios. Optimizing these parameters frequently requires balancing complexity and usability, an area where AI-assisted signal processing has great potential [19].

Use cases across sectors

Biosensor technologies have proven useful across multiple areas of AMR surveillance. They enable the rapid identification of resistant infections, such as methicillin-resistant *Staphylococcus aureus* (MRSA), extended-spectrum β-lactamase (ESBL)-producing Enterobacteriaceae, and carbapenemase producers, directly at the point of care in clinical settings. In the veterinary and agricultural sectors, biosensors facilitate the monitoring of antibiotic residues and resistance genes in livestock, aquaculture, and animal feed [8]. Environmental surveillance also benefits from these technologies, allowing the detection of AMR determinants in wastewater, soil, and surface water, thereby providing insights into community-level resistance and the environmental spread of resistance genes [20]. By integrating laboratory precision with field adaptability, biosensors bridge diagnostic microbiology and public health surveillance. Their continued development, particularly when combined with AI and networked data systems, has the potential to transform global AMR detection and support data-driven biosecurity interventions.

AI integration in biosensing

**Figure 2:**
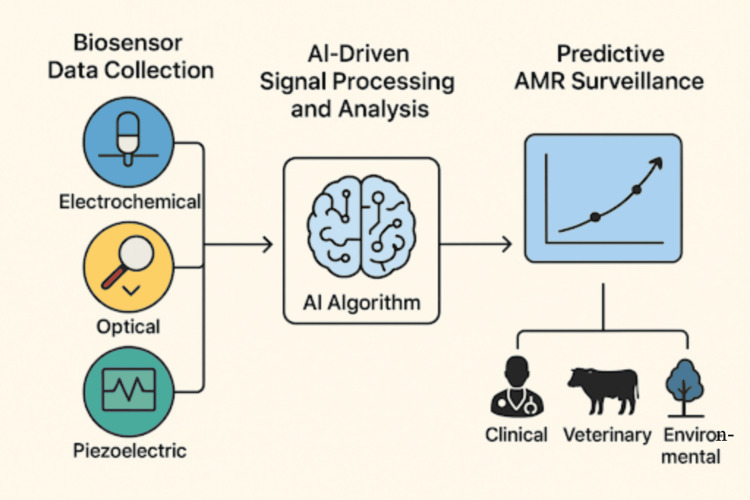
Integration of artificial intelligence (AI) in biosensors for antimicrobial resistance (AMR) detection and surveillance. The graphic shows the AMR monitoring AI-enhanced biosensor integration pipeline. Under the One Health framework, AI-driven signal processing and pattern recognition result in predictive analytics for real-time AMR surveillance in clinical, veterinary, and environmental contexts from electrochemical, optical, and piezoelectric biosensor data. The figure was created by the authors.

The combination of biosensor technology and AI transforms AMR detection and surveillance. Biosensors generate quick and sensitive signals, but signal fluctuation, background noise, and multidimensional dataset interpretation limit their analytical potential. ML and deep learning (DL) algorithms can provide adaptive, autonomous, and data-driven biosensor output interpretation, overcoming these constraints. This integration has enabled smart diagnostics, where biosensors detect and interpret biological data quickly and accurately for real-time AMR surveillance [18,21]. AI-integrated biosensing for AMR detection is conceptualized in Figure 1. It shows how biosensors like electrochemical, optical, and piezoelectric systems collect diagnostic data and analyze it using AI-driven signal processing and ML models to enable predictive AMR surveillance in clinical, veterinary, and environmental domains. Figure 2 shows how AI-enabled biosensors function within an integrative genomics framework under the One Health approach, depicting the interconnected flow of data across clinical, veterinary, and environmental sectors to enable real-time, continuous surveillance of AMR.

Rationale for AI integration

Signal Processing and Noise Reduction

Stochastic fluctuations, environmental interference, and experimental drift affect biosensor signals-electrical, optical, or audio. Traditional statistical methods may not address these noise sources in field-deployable systems. Neural networks and adaptive filtering models can denoise complex data, extract hidden characteristics, and improve detection thresholds. Continuous learning helps these systems adapt to new sensing settings and analytes, boosting robustness [22].

Pattern Recognition for Complex Sensor Outputs

Multimodal biosensing systems produce spectral, electrochemical, and imaging data. AI can identify complex, non-linear patterns that humans cannot. ML methods accurately classify AMR determinants by mapping sensor responses to resistance profiles. Convolutional neural networks (CNNs) can interpret fluorescence and impedance spectra to automatically identify resistance phenotypes from biosensor data [23].

Predictive Analytics for AMR Classification

Through correlation with resistance mechanisms, genotypic markers, and antibiotic susceptibility outcomes, AI improves biosensor prediction beyond detection. Predictive modeling improves clinical and surveillance decision-making by forecasting resistance trends in real time. Biosensors become intelligent systems that can predict AMR emerging patterns due to this analytical depth.

Common AI and ML approaches

AI has been increasingly integrated into biosensing platforms through three main computational approaches. Supervised learning involves training algorithms such as CNNs, support vector machines (SVMs), and random forests on labeled datasets with known AMR outcomes. These models leverage discriminative features to accurately classify new sensor data. SVMs are frequently applied in electrochemical biosensing to differentiate resistant from susceptible bacterial strains, while CNNs are particularly effective for processing image-based or spectral biosensor data [24]. Unsupervised learning employs techniques like k-means clustering and hierarchical clustering to uncover latent structures or detect outliers in biosensor outputs without the need for labeled datasets. These methods are valuable in exploratory AMR research and environmental surveillance, enabling the identification of emerging resistance patterns or previously uncharacterized sensor responses [25]. Additionally, CNNs and recurrent neural network architectures excel at handling high-dimensional biosensor data, making them well-suited for DL applications in image and spectral analysis. DL algorithms can process fluorescence and plasmonic biosensor spectra end-to-end, reducing human bias, improving reproducibility, and providing streamlined, real-time decision support from raw signal acquisition to final categorization [26].

Case studies and emerging applications

Several groundbreaking studies have demonstrated the potential of AI-integrated biosensing for AMR detection. Using ML and electrochemical biosensors, impedance spectra analysis allows bacteria that produce β-lactamase to be identified in less than a minute. MRSA may now be distinguished from susceptible bacteria more quickly and accurately thanks to DL algorithms trained on optical biosensor data. Multiplexed biosensor arrays for environmental monitoring have been interpreted by AI algorithms, enabling One Health surveillance frameworks to track different resistance genes in wastewater samples. These illustrations show the high-throughput, scalable AMR detection potential of AI-enhanced biosensing [27].

Benefits of AI integration

AI integration in biosensing systems offers several key advantages. It enhances accuracy by improving the reliability of resistance detection, classification precision, and signal fidelity. Automation is another benefit, as AI can independently analyze data, reducing the need for expert oversight and enabling real-time monitoring. Scalability is achieved through cloud or edge computing platforms, allowing AI-powered biosensors to support global surveillance and harmonize data across multiple sites. Additionally, AI provides adaptability, as ML models can continuously update and refine their performance in response to new datasets and evolving AMR profiles [17,28].

Challenges and future considerations

Despite its promise, integrating AI into biosensing systems presents several challenges. Data scarcity and standardization remain major obstacles, as large, high-quality, and well-annotated datasets are essential for reliable model training in AMR biosensing research. However, biosensor-generated data are often limited, unevenly distributed, or lack standardized metadata, reducing model robustness. Generalizability and overfitting are additional concerns; models trained on small or homogeneous datasets may perform well in laboratory conditions but fail to adapt across diverse clinical and environmental contexts. The lack of explainability and transparency-the so-called “black box” problem-can further undermine clinician and regulatory trust, limiting adoption in healthcare and surveillance frameworks. Moreover, the computational demands of DL architectures require significant processing power and energy, posing constraints for portable or field-deployable systems. Addressing these issues will require the development of interpretable AI frameworks, standardized performance benchmarks, and open-access AMR biosensor datasets. When these solutions are realized, AI-integrated biosensors could evolve into intelligent surveillance nodes within globally networked health security systems, transforming the landscape of AMR detection [18,29].

Limitations of AI integration

While AI enhances the analytical potential of biosensing, several inherent limitations persist. Data scarcity continues to hinder progress, as the lack of large and diverse datasets constrains model accuracy and reproducibility. This limitation contributes to overfitting, where models optimized for specific datasets lose predictive strength in broader, real-world applications. Additionally, the “black box” nature of many AI algorithms restricts interpretability, making it difficult for clinicians and regulators to validate or trust automated outputs. Advancements in explainable AI (XAI), coupled with open data frameworks and standardized evaluation protocols, are critical to overcoming these challenges. Through such efforts, AI-integrated biosensors can transition from promising prototypes to transparent, scalable, and clinically reliable tools for global AMR surveillance [29].

Integration into surveillance systems

AI-enhanced biosensing technologies in AMR surveillance systems are a major step forward in worldwide resistance monitoring and mitigation. Traditional AMR surveillance relies on centralized laboratory networks, which are accurate but constrained by sluggish data turnaround, geographic limits, and disparate information sharing. Intelligent biosensors can turn surveillance architectures into real-time, distributed, data-rich ecosystems, enabling the One Health concept for human, animal, and environmental health [30].

Surveillance frameworks: a One Health perspective

AMR crosses clinical, veterinary, agricultural, and environmental boundaries, according to the One Health approach. Integrating biosensors allows multisectoral surveillance, where detection data from different ecosystems are combined to understand resistance trends.

Clinical Surveillance

Biosensor-based platforms in hospitals and clinics can feed point-of-care resistance data into infection control systems and national AMR databases.

Veterinary and Agricultural Surveillance

Portable biosensors enable on-site monitoring of antibiotic residues, resistant bacteria, and genes in livestock, aquaculture, and agricultural runoff [31].

Environmental Surveillance

**Figure 3:**
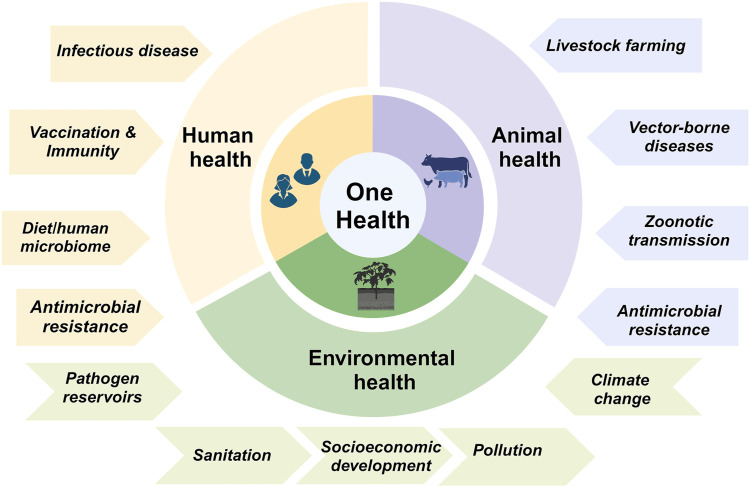
The One Health strategy of monitoring global health. The image is adapted from [32] (Creative Commons Attribution 4.0 International (CC BY 4.0))

Biosensors in wastewater, surface water, and soil identify and quantify resistance determinants, indicating community-level or zoonotic AMR transmission. Incorporating these technologies into cross-sectoral networks, biosensors create a dynamic, continuous surveillance infrastructure that supplements laboratory diagnosis. Figure 3 shows the One Health strategy for monitoring global health, illustrating the interconnected surveillance of human, animal, and environmental health to address issues such as AMR [32].

Real-time data collection and analytics

The convergence of the Internet of Things (IoT), mobile connectivity, and cloud computing has facilitated the development of smart biosensing networks capable of continuous data transmission, storage, and analysis. Wireless modules enable AI-integrated biosensors to report findings in real time to cloud-based surveillance platforms, allowing for immediate data collection and visualization that supports outbreak detection and early warning systems. Geospatial analytics further enhances these networks by linking resistance data to environmental or epidemiological factors, while automated trend analysis using ML models helps identify hotspots and unexpected resistance patterns. This digital integration shifts AMR monitoring from a retrospective approach to a predictive and preventive framework, enabling informed interventions before resistance spreads widely [33].

Applications and case examples

AI-enabled biosensing is being evaluated across diverse surveillance contexts. In wastewater monitoring, biosensor arrays combined with AI algorithms can track AMR genes in municipal wastewater, offering population-level insights into resistance patterns and enabling early outbreak detection. Within hospital settings, biosensors deployed in wastewater or ventilation systems provide real-time tracking of resistant organisms, supporting enhanced infection control measures [20]. On farms, portable electrochemical and paper-based biosensors integrated with ML algorithms have been employed to detect antibiotic residues and resistant bacteria, promoting responsible antimicrobial use and safeguarding the food chain. Collectively, these applications illustrate how networked biosensing systems can enhance the granularity, speed, and reach of AMR surveillance.

Data integration and interoperability

To maximize their value, biosensor-generated data must be compatible with national and international monitoring regimes. Biosensor-derived data can contribute to global resistance mapping and risk assessment by integration with WHO Global AMR Surveillance System (GLASS) and regional One Health networks. Standardized data formats, information annotation, and quality control mechanisms are needed. Cloud-based data management systems with application programming interfaces (APIs) can also enable seamless data exchange across biosensor devices, healthcare systems, and public health databases, turning sensor outputs into epidemiologically actionable insight [34].

Ethical, legal, and logistical considerations

Connected biosensing systems introduce several ethical and operational challenges. Data privacy and security are critical, as real-time transmission of biosensor data necessitates robust encryption and regulatory oversight to protect patient and environmental information. Equitable access to affordable, interoperable biosensor technologies is also essential, particularly for low- and middle-income countries (LMICs), to ensure global AMR surveillance equity. Field calibration and validation are necessary to confirm biosensor reliability across diverse matrices and environmental conditions. Additionally, transparent regulatory and ethical frameworks must be prioritized to balance technological innovation with responsible governance, particularly regarding genetic data and AI-driven decision-making. Addressing these considerations is vital for the trustworthy, equitable, and sustainable deployment of AI-integrated biosensing systems in AMR monitoring [35].

Comparative analysis

Table 1 outlines representative biosensor platform-AI/ML studies for AMR detection or susceptibility inference to help readers navigate the varied field. These examples demonstrate optical, electrochemical, and spectroscopic transduction modalities, AI methods (DL, traditional ML), and application domains (clinical phenotyping, environmental surveillance).

**Table 1:** Representative AI-integrated biosensor studies for AMR detection. AST: antimicrobial susceptibility testing; AI: artificial intelligence; ML: machine learning; CNN: convolutional neural network; SVM: support vector machine; LOD: limit of detection; qPCR: quantitative polymerase chain reaction; AMR: antimicrobial resistance

Study (ref)	Biosensor type	AI/ML approach	Target organism/marker	Key performance/metric	Notes & limitations
Zagajewski et al., 2023 [36]. Deep learning & single-cell phenotyping (AST/rapid AST) (PMC)	Optical/single-cell imaging microfluidics	Deep learning (CNNs/custom networks)	Rapid phenotypic antibiotic susceptibility at single-cell level (various bacteria)	Reported rapid AST with high classification accuracy at single-cell scale; minutes to hours vs. days	High accuracy in controlled samples; needs validation across diverse clinical matrices [36]
Al-Shaebi et al., 2022 [37]. Raman spectroscopy + U-Net for bacterial classification/resistance profiling (ACS Publications)	Raman spectroscopy (label-free)	Deep learning (U-Net for feature extraction + classifiers)	Bacterial species and resistance-associated spectral signatures	High identification accuracy on spectral datasets; improved feature extraction vs. classical methods	Spectral variability and small datasets limit generalizability
Zhang et al., 2025 [38]. Electrochemical ML examples-reviews & platform studies (2024–2025) (MDPI)	Electrochemical impedance/voltammetry sensors	Random forest, SVM, neural nets applied to impedance/time-series features	Enzymatic activity (β-lactamases), gene hybridization surrogates, phenotypic response	Improved sensitivity and LOD when ML used for denoising/classification; reduced false positives	Many studies are proof-of-concept; field robustness and standardization are open issues
Huang et al., 2023 [39]. Wastewater/environmental monitoring (reviews & pilot studies) (ScienceDirect)	Multiplexed sensor arrays/qPCR & sensor hybrids	ML for trend detection, anomaly detection, source attribution	Community-level AMR gene abundance (e.g., bla, mec, and van)	Enables near-real-time trend detection at the population scale in pilot deployments	Standardization, calibration, and linking sensor reads to epidemiological data remain challenging

Trends observed in the literature

Recent advances in AI-integrated biosensing highlight several key trends in AMR surveillance. First, there is a rising convergence of DL with spectroscopy and imaging methods. Label-free optical techniques, such as Raman spectroscopy, SERS, and microscopy, combined with CNN or U-Net architectures, enable rapid phenotyping and species or AMR signature classification without the need for culture. These methods are highly effective for high-dimensional data but remain sensitive to domain shifts caused by variations in sample matrices or instrumentation [40]. Second, point-of-care applications using electrochemical platforms and traditional ML are gaining attention. Electrochemical biosensors paired with feature-based ML algorithms, such as SVMs and random forests, improve signal-to-noise discrimination and allow multiplexed readout interpretation, making them suitable for decentralized antimicrobial susceptibility testing (AST). However, many of these systems remain at the proof-of-concept stage and require extensive field validation [41]. Third, the active translational frontier focuses on environmental and wastewater surveillance. Recent reviews and pilot studies indicate that wastewater provides a feasible aggregate sampling point for population-level AMR monitoring, with AI enhancing trend analysis, hotspot detection, and anomaly identification from multiplexed assays and sensor arrays. Integration with public health dashboards is still in early stages but shows promise [32]. Finally, emerging hybrid techniques that combine phenotypic and genotypic data are being developed. Rapid phenotypic biosensor readouts, targeted molecular assays or sequencing, and AI fusion models together produce richer inferences-such as phenotype prediction alongside potential resistance gene identification, enhancing clinical utility while balancing speed and specificity. This integrated approach is highlighted as a priority for future development [42].

Key gaps and limitations in current studies

The development of AI-integrated biosensing systems for AMR faces several critical challenges. Data volume and diversity are paramount, as robust AI models require large, heterogeneous, and well-annotated datasets spanning clinical, environmental, and device-specific sources. Small, single-site, or instrument-specific datasets increase the risk of overfitting and reduce model generalizability [43]. Moreover, the absence of a common framework or benchmark datasets for evaluating AI-biosensor performance hinders cross-study comparisons and complicates regulatory assessment. Reviews emphasize the need for open, curated datasets and standardized reporting of LODs, sensitivity, specificity, and matrix effects [29]. The “black-box” nature of DL models also impedes clinical and public health adoption, as a lack of interpretability undermines regulatory confidence. Addressing this requires XAI methods and decision-support pipelines that provide transparent, interpretable outcomes [44]. Finally, field validation and robustness remain critical gaps. Many studies report strong laboratory performance, yet comprehensive testing across diverse sample types, geographic regions, and operational conditions is essential for reliable widescale surveillance deployment [45].

Cost-benefit and scalability perspectives

Cost Considerations

Label-free optical systems (Raman, SERS) require expensive optics and stable conditions, while electrochemical and paper-based sensors are cheap to make. AI integration costs money for edge devices, cloud computing, and model maintenance. AI-enabled biosensors can lower per-sample costs by automating interpretation and minimizing lab referrals when amortized across high-throughput deployments (e.g., wastewater networks and hospital wards) [46].

Benefit Calculus

Reduced response time, near-real-time surveillance, and actionable analytics (hotspot detection, early epidemic warnings) are the main benefits. Optimized antibiotic usage, fewer problems, and faster outbreak containment could save healthcare dollars, although they are hard to evaluate but important at population scale B [47].

Scalability Enablers and Barriers

The enablers include low-cost sensor fabrication, edge-AI for on-device inference, standardized APIs for data sharing, and modular cloud platforms for centralized analytics. Barriers include model retraining, data governance limits, inconsistent connections in LMICs, and consumable supply-chain difficulties. Pilot studies imply hybrid deployment models-local edge inference + periodic cloud retraining-can cut bandwidth and privacy risks while maintaining scalability [48].

Summary (comparative analysis takeaways)

The literature shows that AI-integrated biosensors across modalities work, including early successes from spectroscopy/imaging plus DL and electrochemical sensors with traditional ML. However, dataset limits, lack of standardized benchmarks, explainability/regulatory gaps, and insufficient field validation are the main translation challenges. To scale these solutions inside One Health surveillance networks and their cost-benefit potential, strategic investment in curated, open datasets, interoperable reporting standards, and coordinated field trials is essential.

Challenges and limitations

Despite rapid advances in AI-integrated biosensing for AMR detection, various multidimensional hurdles limit translation from lab innovation to real-world surveillance. Biosensing research is difficult, and global health technology ecosystems are unequal; thus, these restrictions span technological, computational, regulatory, and socioeconomic realms. These constraints must be overcome for robust, egalitarian, and sustainable AI-enabled biosensor platform adoption in One Health surveillance systems.

Technical challenges

The diagnostic and surveillance utility of biosensors is closely linked to their technological performance. Sensitivity and LOD have improved through nanomaterial-based transducers and advanced signal amplification methods; however, biosensors often perform inconsistently in complex matrices such as wastewater, soil, and mixed clinical samples. Challenges including biofouling, matrix interference, and variable sample preparation remain significant obstacles [49]. Repeatability and stability are also critical considerations. While laboratory prototypes demonstrate high precision under controlled conditions, field deployments frequently exhibit reduced repeatability due to environmental fluctuations, sensor degradation, and manufacturing variability. Achieving scalability necessitates robust quality-control measures and standardized calibration protocols [50]. Finally, multiplexing and integration capabilities are essential for comprehensive surveillance, enabling simultaneous detection of multiple AMR genes or pathogens. However, multiplexing can introduce signal cross-talk, sensitivity loss, and increased complexity in data interpretation. Despite advances in microfluidic integration and algorithmic signal deconvolution, commercially ready multiplexed AI-biosensor solutions remain limited [51].

Computational challenges

AI enhances the analytical capabilities of biosensing platforms but also introduces increased computational dependency and associated risks. Data quality and quantity are critical for training ML models, which require large, balanced, and well-annotated datasets to generalize effectively. Biosensor datasets are often limited in scale, biased toward specific organisms or sample types, and lack standardized metadata, resulting in overfitting and poor portability across different settings [52]. Model explainability and trust represent another challenge, as many high-performing DL models function as “black boxes,” making them difficult for end-users, clinicians, and regulators to interpret. Given the clinical and policy implications of AMR surveillance, XAI frameworks are essential for transparency and accountability [53]. Data drift and continuous learning further complicate deployment; dynamic changes in resistance profiles and environmental variables can degrade model performance, necessitating periodic retraining and validation with new datasets, which is computationally and logistically demanding [54]. Finally, achieving an effective edge-cloud balance is crucial. Performing AI inference on low-power, edge-based biosensor devices reduces latency and protects privacy but requires lightweight algorithms and hardware optimization. Cloud-based processing allows for more complex analyses but depends on stable connectivity and may pose data security concerns [55].

Implementation and regulatory challenges

One of the major challenges in translating biosensor technologies from laboratory demonstration to practical implementation lies in regulatory, operational, and lifecycle considerations. Regulatory frameworks present significant obstacles, as AI algorithms embedded in diagnostic devices often fall outside existing approval pathways. Few agencies provide clear guidance for adaptive, learning-based biosensors, necessitating harmonization among medical device authorities, data protection agencies, and public health organizations [56]. Quality assurance and biosafety are also critical; biosensors used for AMR detection must incorporate verified disinfection protocols and maintain diagnostic accuracy, particularly when handling pathogens or resistance genes. Mobile devices must comply with stringent biosafety standards [57]. The absence of standardized validation metrics, data-sharing protocols, and reference materials further limits comparability across studies and jurisdictions, while integration with WHO GLASS or national AMR surveillance systems remains fragmented. Finally, lifecycle management poses challenges, as AI components require ongoing maintenance, calibration, and software updates, a need that is often overlooked in implementation planning [58].

Implementation costs and evolving regulatory frameworks for AI-integrated biosensing

This section highlights the economic and regulatory factors influencing the real-world adoption of AI-integrated biosensors. It explains that high-end optical platforms such as Raman and SERS systems involve significant setup and maintenance expenses, while low-cost electrochemical and paper-based sensors offer affordable options for decentralized deployment. AI integration introduces added costs related to data storage, cloud computing, and algorithm maintenance, though automation can offset these over time. The section also emphasizes that regulatory frameworks for adaptive AI diagnostics are still emerging, referencing models such as the U.S. FDA’s Software as a Medical Device (SaMD) framework, the European Union AI Act (2024), and WHO’s GLASS as key examples of evolving standards that promote safety, transparency, and global harmonization in AI-enabled healthcare systems [56].

Socioeconomic and capacity challenges

Technological advancements in AI-integrated biosensing do not automatically translate into global benefits. LMICs, which bear a disproportionate burden of AMR, face infrastructural and financial barriers that limit the adoption of advanced biosensing platforms. Challenges such as power instability, limited internet connectivity, and a shortage of trained personnel further impede implementation. Cost and affordability also constrain widespread deployment, as sensor fabrication, AI integration, and cloud infrastructure remain expensive without dedicated funding or economies of scale; strategies such as open-source hardware, modular software, and public-private partnerships may help reduce expenses. Training and human capital are critical, requiring collaborative education across microbiology, engineering, data science, and public health, yet current institutional structures rarely support such cross-sector expertise. Ethical and equity considerations are equally important, encompassing data ownership, consent, and benefit-sharing, particularly when surveillance involves vulnerable or under-resourced populations. Ensuring fair access to both data and technology is both a moral obligation and a strategic necessity for sustained AMR management [59,60]. Ultimately, AI-integrated biosensors have the potential to transform AMR detection and monitoring, but addressing issues of technical repeatability, computational transparency, regulatory flexibility, and socioeconomic inclusion will determine whether these systems evolve from academic prototypes into globally trusted biosecurity and antimicrobial management tools.

Future perspectives

The combination of AI, biosensing, and digital health will change AMR detection and surveillance. Experimental prototypes will likely give way to fully integrated, intelligent biosensing ecosystems that operate continuously across human, animal, and environmental domains in the next decade. This vision requires synergistic advancements in technology, systems architecture, policy frameworks, and research collaboration.

Technological outlook

Biosensors of the future are expected to be miniaturized, multiplexed, and increasingly autonomous. Next-generation sensing materials and transduction mechanisms, including graphene derivatives, plasmonic nanostructures, and quantum dots, will enhance sensitivity, dynamic range, and multianalyte detection. Hybrid biosensors that combine optical, electrochemical, and acoustic modalities will generate multidimensional data streams, enabling AI systems to infer resistance phenotypes with greater accuracy and robustness. Edge AI and embedded analytics will allow local processing and interpretation of biosensor data, reducing latency and enhancing privacy by shifting computation from the cloud to the device level. With low-power microprocessors and neuromorphic circuits, portable devices will be capable of making real-time decisions even in resource-limited environments. Wearable and implantable biosensors built on flexible, biocompatible platforms will facilitate continuous monitoring of infection biomarkers and antibiotic exposure, offering insights into host-pathogen-drug interactions and supporting personalized antimicrobial therapy as well as early illness detection. Finally, hybrid sensing and automation, through integration with robotic samplers or drones, will enable high-resolution environmental and wastewater surveillance, transforming AMR monitoring into a self-learning, adaptive infrastructure [54,61].

Systems outlook

The global impact of AI-enhanced AMR surveillance will hinge on the effective integration of biosensing data into broader digital ecosystems. By linking biosensor outputs with electronic health records (EHRs), telemedicine platforms, and hospital information systems, rapid feedback loops can be established, connecting detection directly to clinical decision-making. Synergy between genomic and metagenomic data further enhances this capability: combining biosensor-based phenotypic information with sequencing data generates multimodal datasets that can map resistance determinants across populations and ecosystems in near real time. AI algorithms trained on these datasets can uncover hidden transmission pathways and patterns of resistance evolution [62]. For maximal impact, biosensor data should be incorporated into global AMR platforms such as WHO GLASS, Food and Agriculture Organization (FAO)/World Organisation for Animal Health (OIE)/WHO One Health frameworks, and regional digital biosecurity centers. This requires interoperability protocols, standardized metadata, and ontologies for biosensor-derived information [63]. Through these integrated networks, AI-enabled biosensors can detect emerging resistance, support preventive interventions, and enable global risk forecasting, ultimately strengthening AMR surveillance and response across diverse geographic regions.

Policy outlook

The global impact of AI-enhanced AMR surveillance will depend on the seamless integration of biosensing data into digital ecosystems. By linking biosensor outputs with EHRs, telemedicine platforms, and hospital information systems, rapid feedback loops can be established, connecting detection directly to clinical decision-making. Synergy between genomic and metagenomic data further enhances this capability: combining biosensor-based phenotypic information with genomic or metagenomic sequencing generates multimodal datasets that can map resistance determinants across populations and ecosystems in near real time. AI algorithms trained on these datasets can reveal hidden transmission pathways and patterns of resistance evolution [64].

To maximize utility, biosensor data should be incorporated into global AMR platforms, including WHO GLASS, FAO/OIE/WHO One Health frameworks, and regional digital biosecurity centers. This requires the implementation of interoperability protocols, standardized metadata, and biosensor-derived information ontologies [65]. Through these integrated networks, AI-enabled biosensors can detect emerging resistance, support preventive interventions, and enable global risk forecasting, strengthening AMR surveillance and response across diverse geographic regions.

Research and collaboration outlook

Interdisciplinary, open, and internationally coordinated research ecosystems will be essential to drive the advancement of AI-integrated biosensing. Proper AI training and benchmarking require open, annotated biosensor datasets encompassing diverse organisms, resistance mechanisms, and environmental contexts. Establishing open standards for data reporting, model evaluation, and biosensor validation will enhance comparability, reproducibility, and regulatory confidence [66].

Looking ahead, future frontiers involve interdisciplinary collaborations across microbiology, materials science, data science, and policy. Partnerships among academia, public health organizations, and industry can accelerate the translation of laboratory innovations into field-ready applications. Investment in training programs is also critical to bridge the digital-biological divide and cultivate a new generation of experts capable of designing, deploying, and managing intelligent biosensing systems [67].

Together, AI-driven analytics, smart biosensing, and networked monitoring systems represent a paradigm shift in AMR management. These technologies have the potential to transform AMR surveillance from a reactive process to a predictive, preventive, and globally coordinated strategy-forming a cornerstone of 21st-century biosecurity-provided that development is guided by rigorous science, ethical governance, and inclusive collaboration.

## Conclusions

AMR remains one of the most urgent global health threats, undermining vital therapies and destabilizing both human and animal health systems. Traditional detection methods, though scientifically robust, are often constrained by time, cost, and limited accessibility. The integration of AI with biosensing technologies offers a transformative solution, enabling rapid, decentralized detection and intelligent data interpretation. Over the past decade, biosensing research has evolved from simple proof-of-concept devices to advanced, multifunctional platforms capable of identifying infections, resistance genes, and biochemical markers with high sensitivity and specificity. Through AI-driven signal processing, pattern recognition, and predictive analytics, these systems can generate real-time, high-resolution maps of AMR dynamics across clinical, agricultural, and environmental settings, thereby strengthening global biosecurity within the One Health framework. Despite these advances, the field faces critical challenges that must be addressed to achieve global implementation. Persistent technical and computational limitations-including data scarcity, overfitting, and limited model explainability-continue to hinder the deployment of AI-integrated biosensors beyond controlled laboratory conditions. Equally important are policy and socioeconomic barriers, such as ensuring equitable access, affordability, and ethical governance across diverse healthcare settings. Overcoming these obstacles will require open, interoperable datasets, interpretable AI frameworks, and robust international collaboration. Coordinated efforts among researchers, policymakers, and regulatory authorities are essential to ensure that AI-enabled biosensing innovation translates into scalable, safe, and sustainable AMR surveillance solutions.

In summary, AI-integrated biosensors represent a transformative shift toward predictive, participatory, and preventive AMR surveillance. Their success will depend on transparent algorithm design, harmonized regulatory standards, and equitable technology transfer to ensure global accessibility. When responsibly developed and internationally coordinated, these intelligent biosensing systems can evolve into a proactive early-warning network that safeguards antibiotic efficacy, strengthens health system resilience, and advances the shared objectives of global One Health biosecurity.
